# CASE REPORT Superficial Spreading Basal Cell Carcinoma of the Face: A Surgical Challenge

**Published:** 2010-06-21

**Authors:** Yuri T. Jadotte, Navér A. Sarkissian, Helchem Kadire, W. Clark Lambert

**Affiliations:** ^a^Department of Medicine, New Jersey Medical School, Newark, NJ; ^b^Department of Dermatology, New Jersey Medical School, Newark, NJ; ^c^Department of Pathology, New Jersey Medical School, Newark, NJ

## Abstract

**Objective:** We present the case of a white man with a facial nodule suspicious for basal cell carcinoma. **Methods:** Biopsy revealed clusters of basaloid tumor cells with peripheral palisading, consistent with a superficial spreading variant of basal cell carcinoma. **Results:** The patient was treated with Mohs micrographic surgery, with clear margins achieved after the second stage of excision. However, since it was a superficial spreading basal cell carcinoma, this was followed by topical imiquimod treatment. **Conclusion:** Topical chemotherapy with imiquimod or 5-fluorouracil may be valuable alternatives or adjuncts, given the increased likelihood of recurrence after surgical excision of superficial spreading basal cell carcinoma. Mohs surgery is of limited value in the management of superficial spreading basal cell carcinoma because it characteristically shows areas of uninvolved skin between tumor nests.

Basal cell carcinoma (BCC) is the most common skin cancer in white individuals.^1^ Similar to other nonmelanoma skin cancers, its incidence is rising. The nodular, superficial spreading, and infiltrating variants are the 3 most commonly encountered types of BCC in descending order of prevalence.^2^ Superficial spreading basal cell carcinoma (SSBCC) accounts for 15% to 26% of all cases of BCC.^1^ It usually occurs on the trunk and upper extremities but may be seen on the face.^1^,^3^ It appears to be related to acute exposure to sunlight, especially painful sunburns before the age of 20.^4^,^5^

Surgical excision is the most commonly used treatment of BCC.^2^ Mohs micrographic surgery is widely used for high-risk head and neck tumors.^6^ The precise and complete control of peripheral tumor margins that is achieved using Mohs micrographic surgery yields high cure rates with very low recurrence and allows maximal preservation of tissue.^7^,^8^ Given the various phenotypic manifestations of BCC, tailoring the treatment to the appropriate disease variant optimizes therapeutic success. We report on a case of SSBCC of the face. Superficial spreading basal cell carcinoma tends to spread widely with “skip areas” in which no tumor is present, compromising the earlier mentioned treatment approaches.

## CASE REPORT

**Figure 1:**
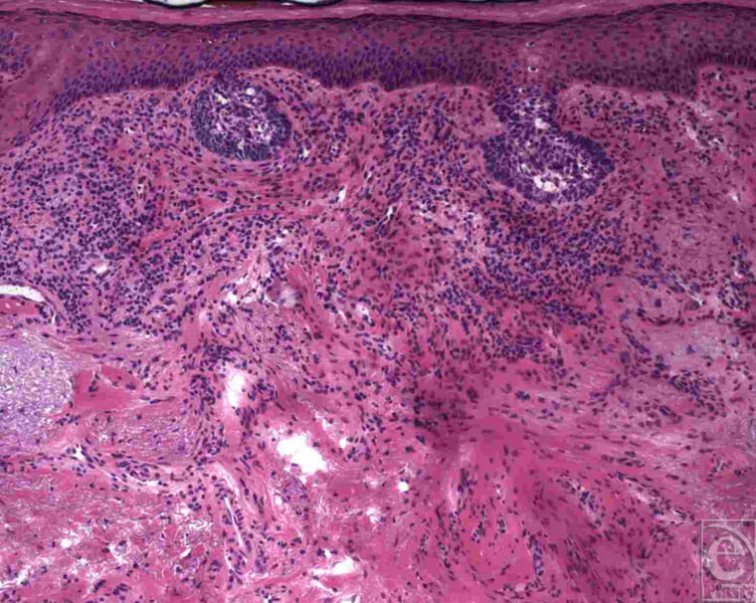
Superficial spreading basal cell carcinoma. A “skip region” can be seen between two nests of tumor cells at the dermal-epidermal junction. Solar elastosis is also evident. (H&E, original magnification ×10).

**Figure 2:**
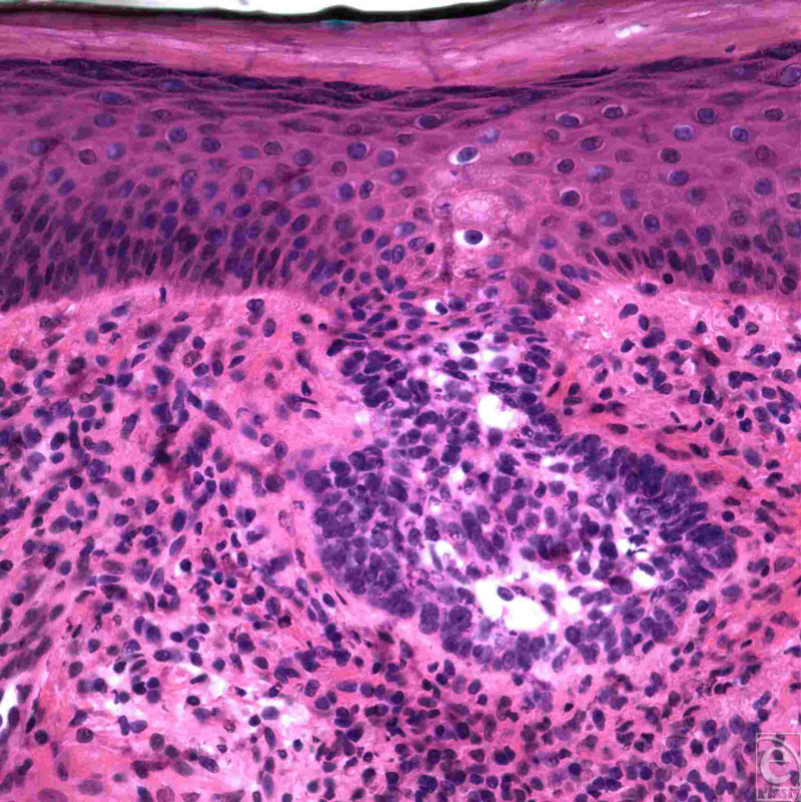
Superficial spreading basal cell carcinoma. A cluster of basaloid tumor cells budding downward from the basal cell layer, limited to the dermal-epidermal junction, is visible here. It is flanked on both sides by tumor-free tissue. (H&E, original magnification ×40).

**Figure 3:**
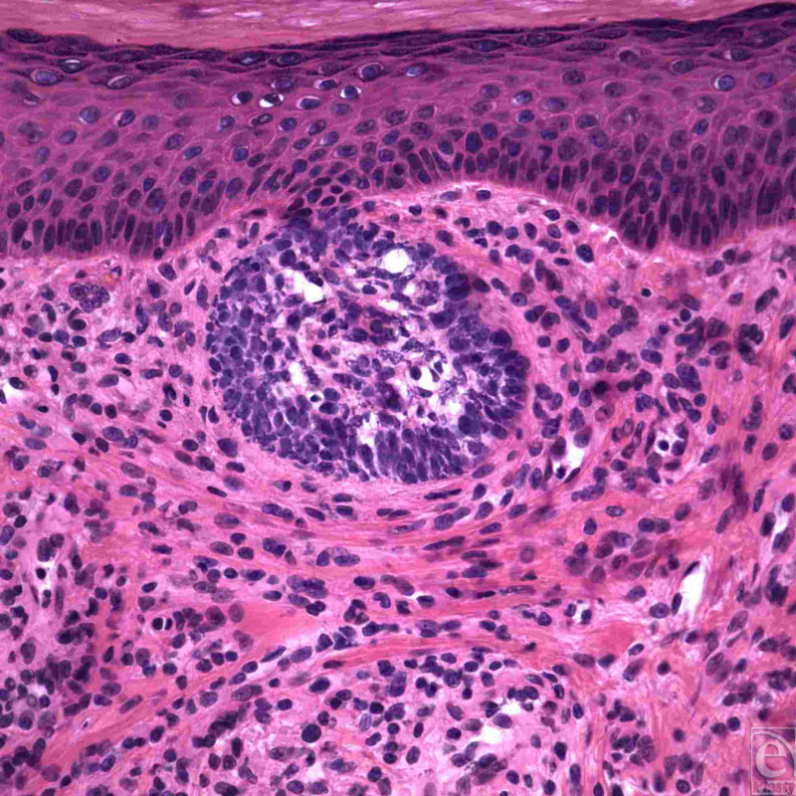
Superficial spreading basal cell carcinoma. These basaloid tumor cells demonstrate hyperchromatism with uniform nuclei and peripheral palisading, but cleft-like spaces are not seen around the nodule. (H&E, original magnification ×40).

A 58-year-old white man with no significant medical history presented with a pearly nodule on the dorsum of the nose. A 5-mm punch excisional biopsy was performed with surgical orientation. Histological analysis of the frozen sections revealed positive margins at 12, 3, and 6 o'clock positions. Multiple nests of tumor cells were seen budding into the dermis from the basal cell layer. They were primarily located at the dermal-epidermal junction. The basaloid tumor cells had hyperchromatic but uniform nuclei and formed a peripheral palisading pattern. However, cleft-like retraction spaces were not seen between the tumor nests and mucinous stroma. Such cleft-like spaces, although characteristic of BCC, are often not seen in SSBCC. The tumor nests spread horizontally. Figures 1 to 3 illustrate these histological findings. A diagnosis of SSBCC was made. A second excisional biopsy was performed, with an additional 2-mm margin taken throughout. Because this was an SSBCC, surgery was followed by treatment with topical imiquimod.

## DISCUSSION

Despite its very high prevalence, BCC is generally a low-grade neoplasm. Although it can be locally invasive and destructive, it rarely metastasizes and is readily amenable to excisional management. However, facial BCC is particularly concerning. It is often found in a cosmetically delicate location. It also has one of the highest recurrence rates of any BCC. Therefore, appropriate diagnosis and therapy are essential.

Superficial spreading basal cell carcinoma of the face is a surgical challenge due to its location, the likelihood of recurrence, and its disseminating pattern. Histologically, SSBCC is characterized by small nests of basaloid cells confined to the epidermis and superficial dermis, originating at the dermal-epidermal junction; there is a horizontal spread usually without deeper dermal involvement.^3^ Superficial spreading basal cell carcinoma also has intermittent regions of normal skin, forming classic “skip regions.” It is also known as “multicentric” or “multifocal” BCC for this reason. Importantly, this unique characteristic makes it difficult to accurately assess the tumor borders after surgical resection with negative margins. This may result in incomplete primary tissue excision and can increase the likelihood of recurrence.^9^

Clinically, SSBCC appears as a red, slightly scaly, and well-demarcated patch. It may present as a solitary patch or multiple patches. It may appear with the characteristic pearly, rolled border with a slightly depressed center. It can resemble a patch of dermatitis and can be confused with eczema, psoriasis, lichen planus, or Bowen's disease.^3^,^10^ Thus, the clinical features alone may not reveal the appropriate diagnosis. Dermoscopy may assist in this endeavor.^10^ Shiny white to red areas; short, fine telangiectasias; and erosions are the hallmark dermoscopic features of SSBCC.^10^

A biopsy is the most reliable diagnostic modality for BCC. Excision is the treatment of choice for BCC. Mohs micrographic surgery is the therapeutic modality of choice for primary and high-risk facial BCCs.^11^ However, because of the nature of SSBCC, the tumors may be inadequately excised despite clear margins. Deep tissue excision may also be unnecessary, as the tumor cells are usually very superficial. Topical imiquimod or 5-fluorouracil may be alternative or adjunct therapies for SSBCC.^12^^-^14

## CONCLUSION

Superficial spreading basal cell carcinoma may recur despite surgical excision with negative margins because of its tendency to produce a mosaic pattern of tumor-free regions. Superficial excision and topical chemotherapy may be preferable in some cases.
